# Genome insights into the pharmaceutical and plant growth promoting features of the novel species *Nocardia alni* sp. nov

**DOI:** 10.1186/s12864-021-08257-y

**Published:** 2022-01-21

**Authors:** Imen Nouioui, Sung-min Ha, Inwoo Baek, Jongsik Chun, Michael Goodfellow

**Affiliations:** 1 Department of Microorganisms, Leibniz Institute DSMZ–German Collection of Microorganisms and Cell Cultures, 38124 Braunschweig, Germany; 2 Department of Integrative Biology and Physiology, University of California Los Angeles, 610 Charles E. Young Drive East, Los Angeles, CA 90095 USA; 3 School of Biological Sciences & Institute of Molecular Biology and Genetics, Seoul National University, Seoul, Korea; 4 ChunLab, Inc, Seoul, Korea; 5 School of Natural and Environmental Sciences, Newcastle University, Ridley Building 2, NE1 7RU Newcastle upon Tyne, UK

**Keywords:** Genome mining, Plant growth promoting properties, Polyphasic taxonomy, Putatively novel antibiotics

## Abstract

**Background:**

Recent studies highlighted the biosynthetic potential of nocardiae to produce diverse novel natural products comparable to that of *Streptomyces*, thereby making them an attractive source of new drug leads. Many of the 119 *Nocardia* validly named species were isolated from natural habitats but little is known about the diversity and the potential of the endophytic nocardiae of root nodule of actinorhizal plants.

**Results:**

The taxonomic status of an actinobacterium strain, designated ncl2^T^, was established in a genome-based polyphasic study. The strain was Gram-stain-positive, produced substrate and aerial hyphae that fragmented into coccoid and rod-like elements and showed chemotaxonomic properties that were also typical of the genus *Nocardia*. It formed a distinct branch in the *Nocardia* 16S rRNA gene tree and was most closely related to the type strains of *Nocardia nova* (98.6%), *Nocardia jiangxiensis* (98.4%), *Nocardia miyuensis* (97.8%) and *Nocardia vaccinii* (97.7%). A comparison of the draft genome sequence generated for the isolate with the whole genome sequences of its closest phylogenetic neighbours showed that it was most closely related to the *N. jiangxiensis, N. miyuensis* and *N. vaccinii* strains, a result underpinned by average nucleotide identity and digital DNA-DNA hybridization data. Corresponding taxogenomic data, including those from a pan-genome sequence analysis showed that strain ncl2^T^ was most closely related to *N. vaccinii* DSM 43285^T^. A combination of genomic, genotypic and phenotypic data distinguished these strains from one another. Consequently, it is proposed that strain ncl2^T^ (= DSM 110931^T^ = CECT 30122^T^) represents a new species within the genus *Nocardia*, namely *Nocardia alni* sp. nov. The genomes of the *N. alni* and *N. vaccinii* strains contained 36 and 29 natural product-biosynthetic gene clusters, respectively, many of which were predicted to encode for a broad range of novel specialised products, notably antibiotics. Genome mining of the *N. alni* strain and the type strains of its closest phylogenetic neighbours revealed the presence of genes associated with direct and indirect mechanisms that promote plant growth. The core genomes of these strains mainly consisted of genes involved in amino acid transport and metabolism, energy production and conversion and transcription.

**Conclusions:**

Our genome-based taxonomic study showed that isolate ncl2^T^ formed a new centre of evolutionary variation within the genus *Nocardia.* This novel endophytic strain contained natural product biosynthetic gene clusters predicted to synthesize novel specialised products, notably antibiotics and genes associated with the expression of plant growth promoting compounds.

**Supplementary Information:**

The online version contains supplementary material available at 10.1186/s12864-021-08257-y.

## Introduction

The actinobacterial genus *Nocardia* [1], the type genus of the family *Nocardiaceae* [2] emend. Zhi et al. [3], has a long and convoluted taxonomic history mainly due to an overreliance placed on morphological properties [4, 5]. The application of polyphasic taxonomic procedures led to marked improvements in the classification of nocardiae and related mycolic acid containing actinobacteria [6]. In general, the genus encompasses aerobic, Gram-stain-positive, acid-alcohol-positive, nonmotile, chemoorganotrophic actinobacteria which form rudimentary to extensively branched substrate hyphae that fragment into coccoid to rod-shaped elements, aerial hyphae may only be visible microscopically; the diamino acid of the peptidoglycan is *meso*-diaminopimelic acid (A_2_pm), the characteristic whole-organism sugars are arabinose and galactose; diphosphatidylglycerol, phosphatidylethanolamine, phosphatidylinositol and phosphatidylinositol mannosides are the major polar lipids; the fatty acids consist of straight-chain, saturated, unsaturated and 10-methyl (tuberculosteric) components; mycolic acids have 46-64 carbon atoms and up to four double bonds; the predominant respiratory quinone is a hexahydrogenated menaquinone with eight isoprene units where the two end ones are cyclized (MK-8[H6-ω-cyclo]) and the DNA G+C content ranges from 63-72 mol% [5, 7].

Many of the 119 *Nocardia* species with validly published names (https://www.bacterio.net/) are recognized using combinations of genotypic and phenotypic properties [7–9]. Most of these taxa are composed of strains isolated from natural habitats but the best-known species contain causal agents of serious suppurative and granulomatous diseases in humans and animals, especially mycetoma and nocardiosis [10–12]. In contrast, *Nocardia vaccinii* produces galls on blueberry plants [13]. Soil is probably the primary reservoir for *Nocardia* strains as they are found in diverse soil types, including acidic forest [14, 15], arid [16], Cerrado [17], karst cave [18], rhizosphere [19, 20] and saline soils [21, 22]. However, they have also been isolated from marine habitats, especially from sponges [23, 24], as well as from the gut of fungus-growing termites [25] and are increasingly being isolated from plant tissue [26], notably from nodules of actinorhizal plants suggesting that they may have a role in promoting plant growth and inhibiting phytopathogens [27, 28]. Two *Nocardia* strains isolated from *Casuarina glauca* nodules induced root nodule-like structures in the original host plant [29].

Nocardiae are an important source of novel antibiotics [30, 31], as exemplified by the production of amicoumacin B from *Nocardia jinanensis* [32], asterobactin from *Nocardia asteroides* [33], brasilicardin A from *Nocardia brasiliensis* [34], nocardicins from *Nocardia uniformis* subsp. *tsuyamanensis* [35] and tubelactomicin A from *Nocardia vinacea* [36]. A comparative survey of nocardial genomes showed that their biosynthetic potential to produce diverse novel natural products is comparable to that of better studied actinobacterial taxa, such as *Amycolatopsis* and *Streptomyces*, thereby making them an attractive source of new drug leads [37]. These researchers showed that *Nocardia* strains from diverse sources, including clinical material, were equally spread across six phylogenetic clades and found that the genomes of the more pathogenic strains were, on average, slightly smaller than those of most of the other genomes (7.4 Mbp against 7.8 Mbp) and contained fewer BGCs (32.5 against 36.5). Similarly, information from the genome of *Nocardia cyriacigeorgica* shows evidence of adaptation from a saprophytic to a pathogenic lifestyle [38].

The present study was designed to establish the taxonomic status of *Nocardia* strain ncl2^T^, isolated from a root nodule of an actinorhizal plant, and to determine its biotechnological and ecological potential. The strain was the subject of a genome-based taxonomic study which showed that it formed a new centre of evolutionary variation within the genus *Nocardia,* the name proposed for this organism is *Nocardia alni* sp. nov. with isolate ncl2^T^ as the type strain. The genomes of *N. alni* and *N. vaccinii* strains contained natural product biosynthetic gene clusters predicted to synthesize novel specialised products, notably antibiotics and genes associated with the expression of plant growth promoting compounds. Statistical comparison between genomic features of the isolate and its taxogenomic neighbours were undertaken to establish any positive correlations between them.

## Materials and methods

### Isolation, maintenance and cultivation

Strain ncl2^T^ was isolated from a root nodule of an *Alnus glutinosa* plant growing in Leazes Park, Newcastle upon Tyne, UK, as described in Ghodhbane-Gtari et al. [27]. The permission to collect the root nodules was obtained and this study complies with local and national regulations. Sterile lobes of harvested nodules prepared using the procedure described by these workers were placed onto BAP agar plates [39] which were incubated until single actinobacterial colonies were detected. One such strain was checked for purity and maintained in 35% (w/v) glycerol at -80°C, as was *Nocardia vaccinii* DSM 43285^T^ obtained from the German Collection of Microorganisms and Cell Cultures (DSMZ). Working cultures of these strains were kept on yeast extract-malt extract agar slopes (International *Streptomyces* Project [ISP] medium 2) [40]. Biomass for the chemotaxonomic analyses carried out on the strains was harvested from ISP2 broths shaken at 200 rpm in baffled flasks for 7 days at 28°C. The harvested cells were washed in distilled water and freeze dried.

### Chemotaxonomic, cultural and morphological properties

Smears from ISP2 agar slopes of the isolate were Gram-stained using Hucker’s modification [41] and the presence of fragmenting branched hyphae sought by light microscopy. The isolate was examined for its ability to grow over a wide range of temperatures (4°C, 10°C, 15°C, 25°C, 28°C, 37°C, 42°C and 45°C) using ISP2 agar as the basal medium. Growth and cultural properties were recorded on GYM (DSMZ medium 65), nutrient agar (NA), peptone-meat extract-glucose agar (DSMZ medium 250) and tryptic soy agar (TSA) after 7 days at 28°C.

The isolate and *N. vaccinii* DSM 43285^T^ were examined for chemotaxonomic properties known to be of value in nocardial systematics [5, 25]. Standard chromatographic procedures were used to establish the diamino acid of the wall peptidoglycan [42], whole organism sugars [43] and polar lipid profiles [44]). Cellular fatty acids were extracted and methylated after Miller [45], as modified by Kyukendall et al. [46], and analysed by gas chromatography (Agilent instrument, model 6890N). The resultant peaks were identified using the Standard Microbial Identification (MIDI) system, version 4.5 and the ACTINO6 database [47]. Mycolic acids were extracted using the procedure described by Minnikin and Goodfellow [48], purified and their profiles determined by gas chromatography (Agilent instrument, model 6890N).

### Genome sequencing

Genomic DNA was extracted from wet biomass of a single colony of isolate ncl2^T^ grown on ISP2 agar for 10 days at 28°C. The extracted DNA was purified and quantified following the protocol of MicrobesNG, Birmingham (UK). Genomic DNA libraries and sequencing were achieved using an Illumina HiSeq instrument and the 250bp paired end protocol, as used in the service provided by MicrobesNG. The draft genome sequence was annotated using the RAST-SEED webserver with default options [49].

### Phylogeny

An almost full length 16S rRNA gene sequence (1523 bp) extracted directly from the draft genome sequence of isolate ncl2^T^ was deposited in the GenBank databases under accession number MZ014381. The resultant gene sequence was compared with corresponding gene sequences of closely related *Nocardia* strains retrieved from the EzBioCloud server [50]. Phylogenetic trees based on single 16S rRNA genes and corresponding genome sequences were inferred using the Type Strain Genome Server (TYG), the high throughput Genome to Genome Distance Calculator (GGDC) webserver of Meier-Kolthoff et al. [51]. Average nucleotide identity (ortho ANI, [52]) and digital DNA-DNA hybridization (dDDH) [53] similarities were determined between the isolate and its phylogenomic neighbours using the ANI calculator from the EZBioCloud (http://www.ezbiocloud.nettools/ani) and the GGDC webservers, respectively. The type strain of *Nocardia casuarinae*, isolated from root nodules of *Casuarina glauca* [27] was included for comparative purposes, as was that of *Nocardia pseudobrasiliensis* which was isolated from a leg abscess of a patient suffering from ulcerative colitis [54].

### Comparative genomic analyses

The genome sequence of isolate ncl2^T^ was compared with that of *N. vaccinii* NBRC 15922^T^ (GenBank accession number BDCC00000000.1), its nearest taxogenomic neighbour, and with those of other phylogenomic relatives, namely *Nocardia jiangxiensis* NBRC 101359^T^ (GenBank accession number BAGB00000000.1), *Nocardia miyunensis* NBRC 108239^T^ (GenBank accession number BDBQ00000000.1), as well as with *N. casuarinae* BMG 51109^T^ (GenBank accession number JAFQ00000000) and *N. pseudobrasiliensis* DSM 44290^T^ (GenBank accession number QQBC00000000.1). These strains were from diverse sources, namely from bud-proliferating galls on blueberry [13], the rhizosphere of goose-grass (*Eleusine indica*), a pine forest soil [14], the root nodule of *Casuarina glauca* and a leg abscess of the patient with ulcerative colitis, respectively. The draft genome assemblies of the strains were annotated using the RAST-SEED webserver [49, 55] with default options.

Genome-based species identification was achieved using the TrueBac ID System v1.92, DB:20190603 [https://www.truebacid.com/] [56] and the algorithm proposed by Chun et al. [57]. For the comparative genomic analyses, homologous regions in the target genomes were determined to query ORFs using the USEARCH program version v8.1.1861, and aligned using a pairwise global alignment [58]. The matched region in the subject contig was extracted and saved as a homolog.

A pairwise gene-to-gene comparison of each genome was conducted using USEARCH and the gene contents among the isolates and related strains compared using the reciprocal homology search tool as described in Chun et al. [59] and Ha et. al [60]. Reciprocal relations are determined if two different genes give bidirectionally top hits to one another. A pairwise orthologous group is defined if the pair of genes has reciprocal relations. The term pairwise orthologous groups (POGs) was coined for these collections of reciprocally linked orthologs. After the initial grouping, partial genes that are grouped due to their short sequence length are targeted for clustering analysis against the POGs using UCLUST (≥95 % identity). The coding sequences (CDSs) were classified into groups based on their roles, with reference to orthologous groups (EggNOG 4.5; http://eggnogdb.embl.de) [61].

Statistical comparisons of the genomic properties of the isolate and those of closely related strains (genome size, digital G+C content, number and median length of codon sequences) were carried out to determine possible correlations between them.

### Phenotypic properties

Strain ncl2^T^ and *N. vaccinii* DSM 43285^T^ were screened for a broad range of phenotypic properties, including their ability to metabolize diverse sole carbon and nitrogen sources, to grow in the presence of several concentrations of sodium chloride, at a range of pH values and in the presence of antibiotics using GENIII microplates and an OMNILOG device (Biolog Inc., Hayward, CA, USA), as described previously [8]. The resultant data were analysed using version 1.3.36 of the OPM package [62, 63]. They were also tested for their ability to produce niacin, arylsulfatase after 3 days [64], and to reduce tellurite [65]. All of these tests were carried out in duplicate using a standard inoculum. Enzymatic and additional metabolic properties of the strains were determined using API-ZYM kits and the protocol provided by the manufacturer (Biomerieux, France).

## Results and discussion

### Chemotaxonomic, cultural and micromorphological properties

Isolate ncl2^T^ showed a range of phenotypic properties consistent with its classification in the genus *Nocardia* [5]. It is aerobic, Gram-strain-positive and forms extensively branched substrate hyphae and aerial hyphae that fragment into coccoid to rod-like elements. Beige-pinkish aerial mycelia were formed on DSMZ 65; ISP2 and TSA agar after 10 days at 28°C and 37°C (Fig. S1). The strain grew well on DSMZ 65 and ISP2 agar at 28°C and 37°C, but not at 4°C, 10°C, 15°C, 25°C, 42°C or 45°C, nor did it grow on nutrient agar. It contained *meso*-A_2_pm as the diamino acid of the peptidoglycan, arabinose, galactose and glucose as cellular sugars; mycolic acids with 42-62 carbon atoms; the major fatty acids (>10%) were C_16.0_ and C_18.1_ ω9c (Table S1) and the polar lipid pattern consisted of diphosphatidylglycerol (DPG), phosphatidylethanolamine (PE), phosphatidylinositol (PI), phosphoglycolipid (PGL), and unidentified phospholipids (PL1-2), aminolipid (AL) and lipids (L1-2) as shown in Fig. S5.

### Phylogeny

**Fig. 1:**
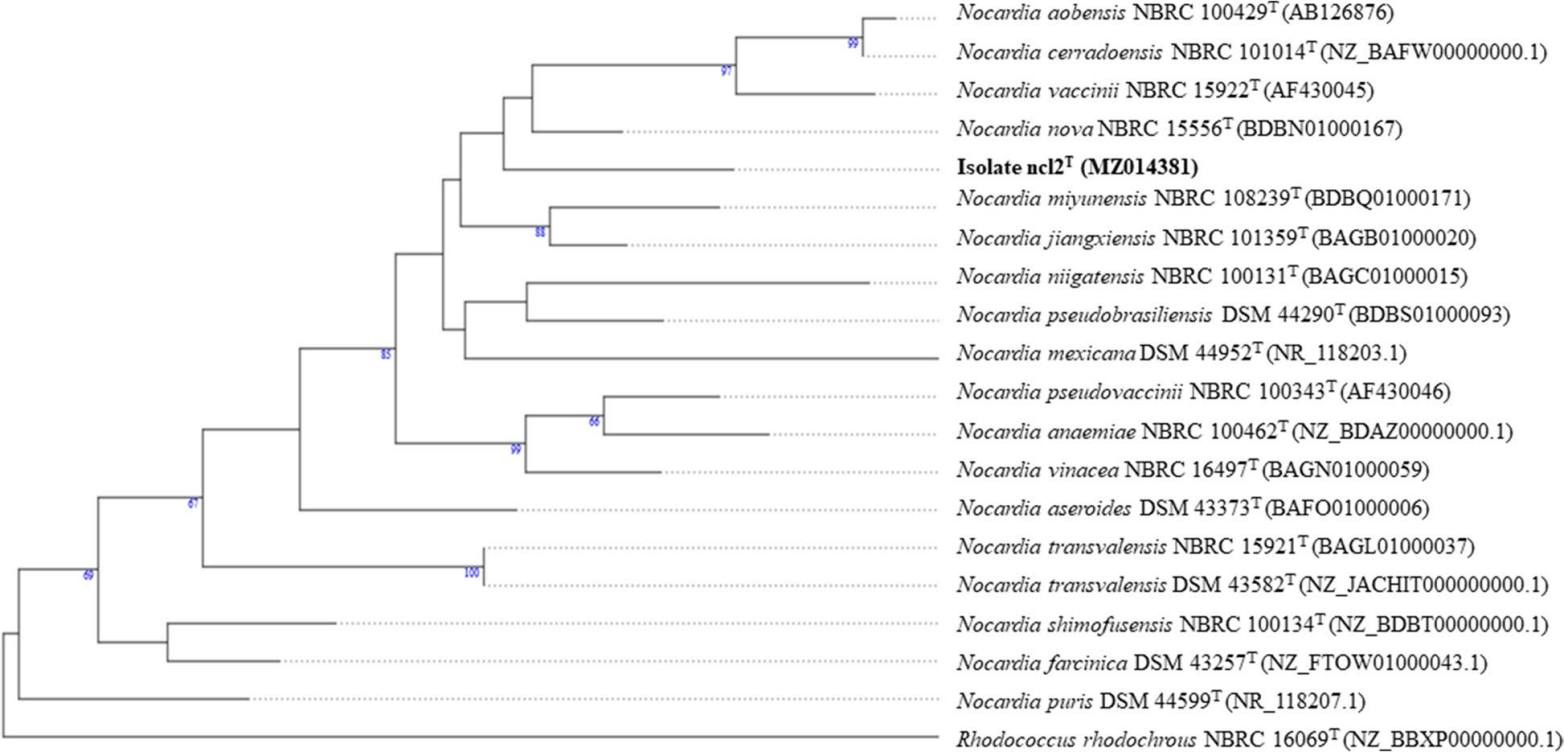
Maximum-likelihood tree based on almost complete 16S rRNA gene sequences showing relationships between isolate ncl2^T^ and the type strains of closely related *Nocardia* species. The numbers at the nodes are bootstrap support values greater than 60%. The root position of the tree was determined using *Rhodococcus rhodochrous* NBRC 16069^T^; the type strain of the type species of the genus *Rhodococcus*

The phylogenetic tree (Fig. 1) shows that strain ncl2^T^ forms a distinct branch in the *Nocardia* 16S rRNA gene tree which lies between lineages composed of the type strains of *Nocardia nova* [66], *Nocardia aobensis* [67], *Nocardia cerradoensis* [17], and *N. vaccinii*, and *N*. *jiangxiensis* and *N. miyunensis,* respectively*.* The close relationship between the *N*. *jiangxiensis* and *N. miyunensis* strains has been recorded repeatedly [14, 24, 25, 68, 69]. Isolate ncl2^T^ was most closely related to *N. nova* NBRC 15556^T^, these strains shared a 16S rRNA gene sequence similarity of 98.6%, which corresponds to 20 nucleotides differences. It also showed relatively low sequence similarities with the *N. aobensis* (97.2%), *N. cerradoensis* (97.0%), *N*. *jiangxiensis* (98.4%), *N. miyunensis* (97.8%) and *N. vaccinii* (97.7%), these relationships were not supported by high bootstrap values (Fig. 1).

**Fig. 2:**
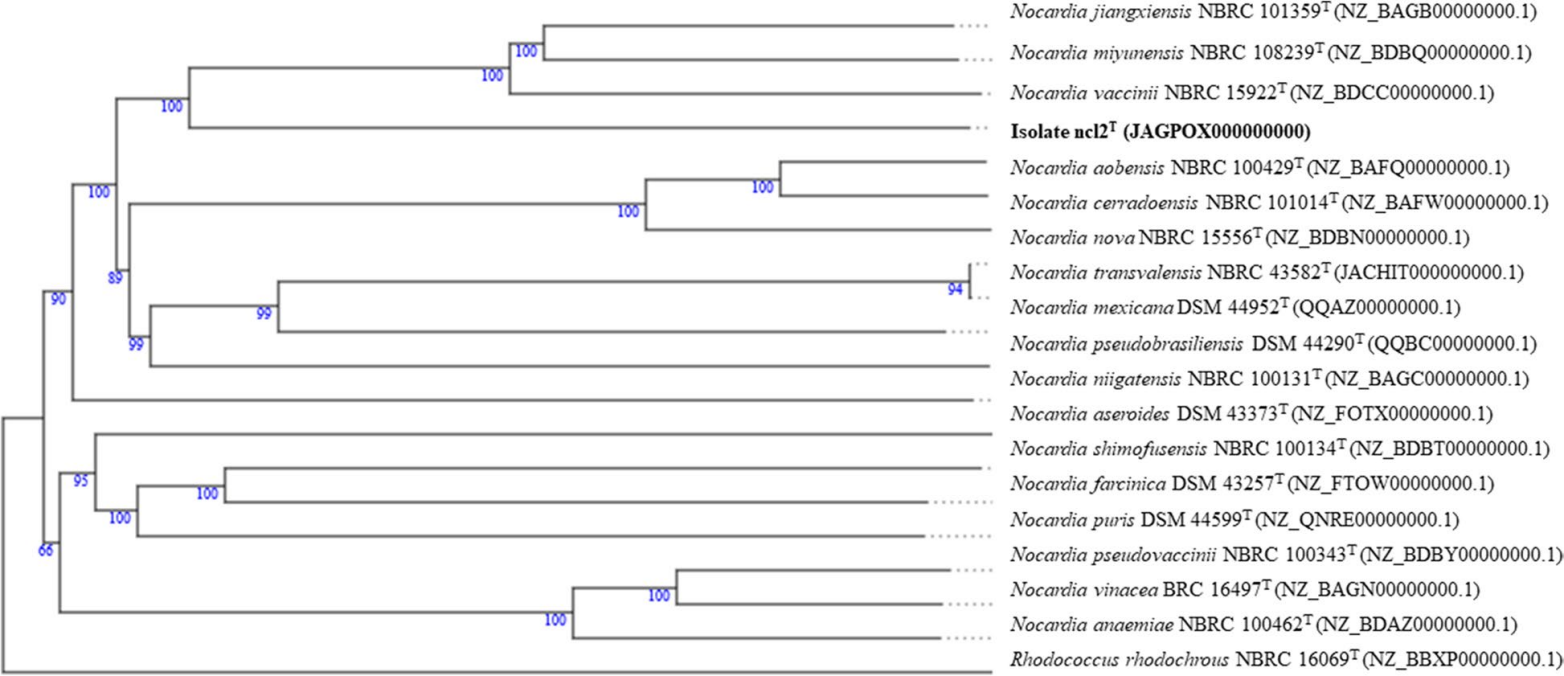
Phylogenomic tree showing relationships between isolate ncl2^T^ and the type strains of closely related *Nocardia* species using the TYG webserver. The numbers at the nodes are bootstrap values greater than 60%

Greater confidence can be placed in the topology of phylogenomic tree when compared against corresponding 16S rRNA gene tree as the former is based on millions, not hundreds, of unit characters [70]. The phylogenomic tree (Fig. 2) shows that isolate ncl2^T^ forms a distinct branch that is most closely related to an evolutionary group composed of the type strains of *N*. *jiangxiensis, N. miyunensis* and *N. vaccinii,* a relationship supported by a 100% bootstrap value. The members of this lineage form a subclade next to a well-supported taxon composed of the type strains of *N. aobensis*, *N. cerradoensis* and *N. nova.*

The recommended threshold used to distinguish between closely related strains based on ANI and DDH values are 95% to 96% and 70%, respectively [57, 71]. Table 1 shows that on this basis isolate ncl2^T^ can be distinguished from its closest phylogenomic neighbours and from the type strains of *N. casuarinae* and *N. pseudobrasiliensis*. It is also clear that it is most closely related to the *N. jiangxiensis, N. miyunensis* and *N. vaccinii* strains although the shared ANI and dDDH similarities are low falling within the range 80.2% to 80.7% and 24.4% to 24.9%. respectively.

**Table 1:** Average nucleotide identity (ANI) and digital DNA-DNA hybridization (dDDH) values between strain ncl2^T^ and its closest phylogenomic neighbours and with the type strains of *N. casuarinae* and *Nocardia pseudobrasiliensis*

Species	Strain designation	dDDH values	ANI values
*N. aobensis*	NBRC 100429^T^	22.4	77.9
*N. casuarinae*	BMG 51109 ^T^	23.0	78.9
*N. cerradoensis*	NBRC 101014^T^	22.4	77.8
*N. jiangxiensis*	NBRC 101359 ^T^	24.6	80.6
*N. miyunensis*	NBRC 108239 ^T^	24.9	80.7
*N. nova*	NBRC 15556^T^	22.4	77.9
*N. pseudobrasiliensis*	DSM 44290^T^	22.4	78.7
*N. vaccinii*	NBRC 15922^T^	24.4	80.2

#### Genome features

Key genomic features of the isolate and the associated reference strains are shown in Table 2 The *in silico* G+C content of the strains fall within a narrow range, namely 66.7 to 68.9%. In contrast, the corresponding genomes show more variation ranging from 8.4 up to 10.5 Mbp in size. The isolate is most closely related to *N. vaccinii* DSM 43285^T^, as these strains have genomes of a similar size (9.9 Mbp and 9.2 Mbp, respectively), very similar dG+C values (67.0% and 66.7%, respectively), and a similar number of CDCs (8969 and 8502) and tRNA genes (49). In contrast, the *N*. *jiangxiensis* and *N. miyunensis* strains have larger genomes, namely 10.45 and 10.52 Mbp, respectively, and a similar, albeit larger, number of CDS; these results underpin the close relationship between these strains as found in 16S rRNA gene sequence analyses*.* The *N. pseudobrasiliensis* strain, a representative of a species associated with invasive human diseases [54], has the smallest genome, thereby providing further evidence that genome sizes of clinically significant nocardiae are lower than corresponding results from non-pathogenic strains [37]. It is also interesting that the type strain of *N. casuarinae*, which induces root nodule formation in *C. glauca,* has a genome size similar to that of *N. pseudobrasiliensis* DSM 44285^T^*.*

**Table 2:** Genomic features of isolate ncl2^T^ (1) and the type strains of *N. vaccinii* (2), *N. miyunensis* (3), *N. jiangxiensis* (4), its closest phylogenetic relatives, and with those of *Nocardia casuarinae* (5) and *N. pseudobrasilensis* (6)

Genomic features	1	2	3	4	5	6
Genome size (Mbp)	9.9	9.2	10.5	10.4	8.8	8.4
Digital G+C content	67.0	66.7	66.9	66.7	68.9	67.3
No. of rRNA genes	12	4	4	3	9	8
No. of tRNA genes	49	49	49	49	56	51
No. of CDSs	8969	8502	9567	9695	7746	7949
Median length of CDS (bp)	831	816	831	822	819	822

A comparison of the taxogenomic features (genome size, digital GC content, number of CDSs, median length of CDS) of strain ncl2^T^ and the five associated reference strains shows that the number of CDSs is positively correlated with genome size and with the coefficient of determination (R2=0.94), this means that 94% of the data points support the predicted regression lines (y=c[176]+c[896]x), shown in Fig. S2. The frequency plot of the pan genome orthologous groups (POGs) of the strains highlight POGs involved in amino acid and carbohydrate metabolism, information storage and processing (eg. recombination and replication) and cellular processes and signaling (Fig. S3). However, no clear correlation was found between genome size and the number of orthologous gene groups though the frequency plots of the POGs for the genomes of the *N. casuarinae, N. jiangxiensis, N. miyunensis* strains were similar with little evidence of quantitative variation.

Comparison of the functional categories between genes in the core and pan genomes using COG/EggNog software gives results in good agreement with those found in the SEED analysis. Apart from genes with unknown function and unassigned categories, the core genomes are composed mainly of genes involved in amino acid transport and metabolism, energy production, and conversion and transcription, as shown in Fig. S4a. However; the pan genome of the strains also contain genes associated with carbohydrate metabolism, clustering based systems and the metabolism of amino acids and derivatives, as presented in Fig. S4b. Around 75% of the core genomes are composed of genes assigned to defined categories whereas less than 40% of those in the pan genomes are associated with functional categories based on COG and SEED pathways. When the strains were examined for strain specific CDSs the highest number were for strain ncl2^T^ with 2162 and the *N. pseudobrasiliensis* DSM 44290^T^ with 2187. The highest similarity was between the pan genome of strain ncl2^T^ and its closest taxogenomic neighbour, *N. vaccinii*, then by the *N. jiangxiensis, N. miyunensis* strains which were isolated from acidic soil; no correlation was found between the genome gene contents of the strains and the habitats from which they were isolated.

#### Phenotypic properties

Identical results were obtained for nearly all of the duplicated cultures included in the phenotypic tests. Table 3 shows that the isolate can be distinguished from *N. vaccinii* DSM 43285^T^ as it produced niacin, utilized L-alanine and L-serine and an extensive range of organic compounds, including β-gentiobiose, dextrin, D-maltose, D-mannose, pectin, D-raffinose, L-rhamnose, sucrose, D-trehalose and D-turanose, was resistant to fusidic acid, lincomycin, minocycline, troleandomycin and vancomycin, and grew in the presence of lithium chloride, guanidine hydrochloride, sodium bromate, sodium chloride (4% and 8%), tetrazolium blue and tetrazolium violet. In contrast, only the *N. vaccinii* strain utilized α-*keto*-glutaric acid, L-malic acid, mucic acid, propionic acid and bromo-succinic acid, it also showed greater activity in the API-ZYM tests.

**Table 3:** Phenotypic, enzymatic and biochemical properties that distinguish strain ncl2^T^ from *Nocardia vaccinii* DSM 43285^T^

**Characteristics**	**Isolate ncl2**^**T**^	***N. vaccinii* DSM 43285**^**T**^
**GEN III Biolog microplate tests**		
** Sugars:**		
D-Cellobiose, D-galactose, β-gentiobiose, D-maltose, D-mannose, D-raffinose, L-rhamnose, sucrose, D-trehalose, and D-turanose	+	-
** Polymers:**		
Dextrin, pectin	+	-
** Tolerance to:**		
Lithium chloride, guanidine hydrochloride, sodium bromate, sodium chloride (4% and 8%), tetrazolium blue, and tetrazolium violet	+	-
**Sugar alcohols:**		
D-Arabitol	+	-
D-Mannitol	-	+
**Oxidation of amino acids:**		
L-Alanine, L-serine	+	-
L-Aspartic acid	-	+
**Resistant to:**		
Fusidic acid, lincomycin, minocycline, troleandomycin, and vancomycin	+	-
**Sugar acids:**		
L-Galactonic acid-γ-lactone, andD-galacturonic acid	+	-
**Organic acids**		
γ-amino-n-Butyric acid, α-hydroxy-butyric acid, L-lactic acid, D-lactic acid methyl ester, *p*-hydroxy-phenylacetic acid, methyl pyruvate, and quinic acid,	+	-
α-*keto*-Glutaric acid, L-malic acid, mucic acid, propionic acid, and bromo-succinic acid,	-	+
**API ZYM tests:**		
Alkaline phosphatase, α-chymotrypsin, cystine aminopeptidase, esterase (C4), esterase lipase (C8), lipase (C14), trypsin, and valine aminopeptidase	-	+
**Other tests:**		
**Niacin**	+	-

+, positive result; -, negative result

Both strains reduced potassium tellurite and gave positive results for acid phosphatase, β-galactosidase α-glucosidase; oxidized, D-glucose, D-gluconic acid, glycerol, and *myo*-inositol (sugars); acetic acid, acetoacetic acid, butyric acid, β-hydroxy-butyric acid, α-*keto*-butyric acid, citric acid, and D-saccharic acid, (organic acids); L-arginine, L-glutamic acid and D-serine #2 (amino acids); were resistant to nalidixic acid, and rifamycin sv (antibiotics); and grew in presence of potassium tellurite, and Tween 40 (inhibitory compounds); and in the presence 1% sodium lactate, sodium chloride (1%) (salts) and at pH 5- 6. The strains gave negative results for arylsulfatase after 3 days, D-fucose, L-fucose, D-fructose, D-fructose-6-phosphate, *N*-acetyl-D-galactosamine, *N*-acetyl-D-glucosamine, D-glucose-6-phosphate, glucuronamide, 3-*O*-methyl-D-glucose, β-methyl-D-glucoside, α-D-lactose, D-melibiose, D-salicin, D-sorbitol, and D-stachyose (sugars); D-gluconic acid, D-malic acid, N-acetyl-neuraminic acid, and L-pyroglutamic acid (organic acids), D-aspartic acid, glycine-proline, L-histidine, and D-serine #1 (amino acids); inosine (nucleoside); aztreonam (antibiotic); gelatin (polymer), niaproof, and sodium formate (inhibitory compounds); α-fucosidase; α-galactosidase, N-acetyl-β-glucosaminidase; β-glucuronidase, and α-mannosidase (API-ZYM tests)

Strains ncl2^T^ and *N. vaccinii* NBRC 15922^T^ can also be distinguished using chemotaxonomic and genomic traits. They showed qualitative and quantitative differences in fatty acid profiles, as exemplified by the presence of a trace of C_17:1_ω9c and a high proportion of C_18:0_10-methyl in the *N. vaccinii* strain and a higher proportion of C_18:1_ω9c in the isolate (24.4% against 9.6%) (Table S1). These properties distinguish them from the type strains of *N. jiangxiensis* and *N. miyunensis* as shown in Table S1. Further, only the isolate contains glucose in whole cell hydrolysates and unidentified aminolipid and glycolipid in its polar lipid profile, unlike *N. vaccinii* DSM 43285^T^ (Fig. S5). The isolate contains mycolic acids ranging from 42 to 62 carbons with predominant amounts of C42 and C44 whereas the *N. vaccinii* strain has mycolic acids ranging from 52 to 62 carbons with C54, C56, and C58 as major components. Both strains have *meso*-A_2_pm as the diamino acid of the wall peptidoglycan, arabinose and galactose as diagnostic whole cell sugars and C_16:0_ as the major fatty acids. Furthermore, the genome size of the isolate is larger than that of the *N. vaccinii* strain (9.9 against 9.2 Mbp).

#### Specialized metabolite biosynthetic gene clusters

Antismash 5.0 [72] predicts NP-BGCs based on the percentage of genes from the closest known bioclusters which show BLAST hits to the genomes of the strains under consideration. The genomes of strain ncl2^T^ and *N. vaccinii* NBRC 15922^T^ contained 36 and 29 well-defined bioclusters that are predicted to encode for a broad range of specialized metabolites albeit with low levels of gene identity, as shown in Table S2. The genomes of the strains are well equipped to synthesize non-ribosomal peptide syntethases, type I polyketides, ribosomally synthesized and post-translationally modified peptides, as well as betalactone (3% gene similarity) and carotenoid-like terpene (18% gene similarity) compounds. They have the genetic capacity to produce products most closely related to himastatin (3% gene similarity), an antitumor antibiotic produced by *Streptomyces hygroscopicus* [73], steffimycin D (8% gene similarity), which was initially produced by a *Streptomyces* strain and inhibits ras-oncogen expressed cells [74], and teicoplanin, a product of a *Streptomyces* strain that inhibits growth of Gram-positive bacteria, including *Enterococcus faecalis* and methicillin-resistant *Staphylococcus aureus* (MRSA) strains [75]. The strains also contain bioclusters predicted to synthesise arylpolyene-like compounds that are structurally and functionally similar to caretonoids [76] and which show antimicrobial and antioxidant activity [77]. They also have bioclusters predicted to encode for ectoine (100% gene similarity), a protective molecule which enables bacteria to survive extreme conditions [78].

The genome of strain ncl2^T^, unlike that of *N. vaccinii* NBRC 15922^T^, contains presumptive NP-BGCs associated with the synthesis of amycolamycin A and B (2% gene similarity), type I polyketides that are cytotoxic for breast cancer cell lines [79], pepticinnamin E (6% gene similarity) which has the potential to treat cancer and malaria [80] and echosides A, B, C, D and E (11% gene similarity) and stambomycin A to D (36% gene similarity),which are antitumor antibiotics [81]. Strain ncl2^T^ also contains bioclusters predicted to encode for the antitubercular polyketides atratumycin (7% gene similarity) and capreomycin A and B (6% gene similarity), which are produced by ‘*Streptomyces aratus’* and ‘*Streptomyces capreolus’,* respectively [32, 82]. Additional presumptive bioclusters are linked to the synthesis of lasolocid (3% gene similarity), a betalactone produced by ‘*Streptomyces lasaliensis’*, which has antibacterial and coccidiostatic properties [83] and tiacumicin B (3% gene similarity), a type I polyketide active against nosocomial diarrahea caused by *Clostridium difficile* [84]. Other bioclusters are predicted to encode for the antifungal agents fengycin (3% gene similarity) and nystatin (31% gene similarity) that are produced by *Bacillus subtilis* and *Streptomyces noursei* strains*,* respectively [85–87]*.*

The genome of the *N. vaccinii* strain harbours several bioclusters absent from that of strain ncl2^T^, including ones predicted to encode for polyketides (Table S2). These bioclusters include cyphomycin (2% gene similarity), which is produced by a *Streptomyces* strain and is used to control multidrug resistant fungal pathogens [88] and caniferolides A- D, that are synthesized by *Streptomyces caniferus* and inhibit the growth of *Candida albicans* and *Aspergillus* spp. [89]; caniferolide A has been used to treat Alzheimer's disease [90]*.* Further, the *N. vaccinii* strain has the genetic capacity to synthesise cremimycin MJ635-86F5 and tetarimycins A and B, these antibiotics are produced by *Streptomyces* strains and show activity against MRSA strains [91, 92]*.* In addition, the strain contains a presumptive biocluster associated with the expression of leinamycin (2% gene similarity), a betalactone terpene produced by *Streptomyces atroolivaceus* which shows antibacterial and antitumor activity [93]*.*

It can be concluded that strain ncl2^T^ and *N. vaccinii* NBRC 15928^T^ have genomes rich in NP-BGCs, notably ones predicted to express for putatively novel polyketide and non-ribosomal peptide compounds thereby providing further evidence that nocardiae are a potentially prolific source of new bioactive compounds [37]. It is particularly interesting that these strains have the capacity to synthesise antifungal and antibiotics given their association with plant tissues. Clearly, nocardiae should feature more prominently in natural product discovery campaigns.

#### Plant growth promoting properties

Comparative genome mining of strain ncl2^T^ and the type strains of *N*. *jiangxiensis*, *N. miyunensis* and *N. vaccinii*, its closest phylogenomic neighbours, revealed the presence of genes associated with direct (eg. phosphate solubilization, phytohormone production) and indirect (eg. lytic enzyme and siderophore production) mechanisms that promote plant growth. *Nocardia casuarinae* BMG51109^T^ and *N. pseudobrasiliensis* DSM 44290^T^ were included in these analyses to represent taxa isolated from plant and clinical sources, respectively [27, 54].

Microbes have a pivotal role in making phosphorus available to plants [94] either enzymatically [95] or by producing organic acids and siderophores and other molecules that solubilize inorganic phosphate [96, 97]. The genome of all of the strains, apart from that of the *N. pseudobrasiliensis* DSM 44290^T^, contained genes associated with phosphate regulation and metabolism (Table S3). These included gene *ppx*-*gpp*A, which is responsible for the solubilization of inorganic polyphosphate [98] and gene *pst*S which encodes for phosphate binding protein PstS that is involved in the production of the phosphate ABC transporter [99]. The *pst*S gene was not detected in the genome of the clinical isolate thereby suggesting a possible correlation between the environmental origin of the other strains, namely soil and plant tissues, and phosphate metabolism. The genome of all of the strains contained gene *sen*X3 which is associated with the production of histidine kinase, a high affinity phosphate transporter which has a role in controlling the phosphate regulon [100].

Phytohormones have a central role in plant growth [101], notably indole -3-acetic acid (IAA) and ethylene; the levels of these and other auxins in plants can be regulated by soil microorganisms able to synthesize them [102]. The genome of all of the strains contained genes encoding for indole-3-glycerol phosphate synthase, the precursor of IAA in the tryptophan biosynthetic pathway in plants [103]. They also contained genes encoding for other components of this pathway, including anthranilate phosphoribosyl transferase (*trp*D), anthranilate synthase (*trp*E), and aminase (*trp* A and B) [104]. Similarly, gene *trp*F, which is associated with the synthesis of anthranilate phosphoribosyl transferase, was present in the genomes of all of the strains, apart from *N. pseudobrasiliensis* DSM 44290^T^. Genes *pdx*l and *aad*, which encode for pyridoxine 4-dehydrogenase and aryl-alcohol dehydrogenase (NADP (+)) and are involved in auxin signaling pathways, were found in the genomes of strain ncl2^T^, *N. jiangxiensis* NBRC 101359^T^, *N. miyunensis* NBRC 108239^T^ and *N. casurinae* BMG51109^T^ (Table S3). In contrast, the genomes of all of the strains contained genes associated with tricarboxylic acid biosynthesis, as shown in Table S3. However, only the genome of strain ncl2^T^ contains gene *acc* that encodes for 1-aminocylopropane-1-carboxylatedeaminase, an ACC deaminase which reduces toxicity due to high levels of ethylene in plants caused by plant growth promoting rhizobacteria. This enzyme also regulates ethylene levels produced by the plant by converting ACC to ammonia and α-ketobutyrate [105, 106].

Plant growth promoting microorganisms can also enhance plant growth by modulating biotic stress as they can decrease, neutralize or prevent infections caused by phytopathogens by synthesizing antibiotics and lytic enzymes [107]. The genomes of all of the strains were equipped with genes associated with the production of chitinases and glucoamylases, as shown in Table S3. They also contained genes involved in the biosynthesis of antibiotics, as exemplified by *fab*G, *bacC*2 and *hdh*A which express for 3-oxoacyl-[acyl-carrier-protein] reductase, bacitracin synthase and 7-alpha-hydroxysteroid dehydrogenase which play a role in the biosynthesis of pentalenolactone, bacitracin and clavulanic acid, respectively [108–110]. Further, the genomes of all of the strains, apart from *N. pseudobrasiliensis* DSM 442990^T^, contained gene *aua*J which encodes for the epoxidase LasC that is involved in the synthesis of lasalocid, a polyether antibiotic [111]. In contrast, only strain ncl2^T^ contained gene *tcm*O which expresses tetracenomycin polyketide synthesis 8-O-methyl transferase, a gene is associated with tetracenomycin biosynthesis [112].

It can be concluded that while strain ncl2^T^ is most closely related to the type strains of *N. jiangxiensis, N. miyunensis* and *N. vaccinii*, it can be distinguished from them as it forms a distinct branch in the phylogenomic tree, has a distinct fatty acid profile and shares low ANI and dDDH values with them. Genomic features, notably genome size and CDS numbers, show that the strain is most closely related to *N. vaccinii* NBRC15992^T^, but can be distinguished from the latter by a wealth of chemotaxonomic, genomic and phenotypic data. It is, therefore, proposed that strain ncl2^T^ should be recognized as a new species within the genus *Nocardia* for which the name *Nocardia alni* sp. nov. is proposed.

### Description of *Nocardia alni* sp. nov.

*Nocardia alni* (al’ni L. gen. fem. n. *alni*, of *Alnus* (a genus name), referring to the source of the strain, a root nodule of *Alnus glutinosa*)

Aerobic, Gram-stain-positive, nonmotile actinobacterium that forms an extensively branched substrate mycelium and aerial hyphae which fragment into coccoid to rod-like elements. Beige pink aerial hyphae are formed on DSMZ 65, yeast extract-malt extract and tryptic soy agar. Grows from pH 5-7.5 (optimally at pH 7), from 28 to 37°C and in the presence of up to 8% w/v sodium chloride. Produces niacin, reduces potassium tellurite, but is arylsulfatase negative after 3 days. Additional phenotypic properties are shown in Table 3. The diamino acid of the peptidoglycan is *meso*-A_2_pm, the whole cell sugars are arabinose, galactose and glucose and the predominant fatty acids are C_16:0_ and C_18:1_ω9c. Mycolic acids have 42 to 62 carbon atoms and the polar lipids are diphosphatidylglycerol, phosphatidylethanolamine and phosphatidylinositol, unidentified phosphoglycolipid, phospholipids, lipids, an aminolipid and a glycolipid. The genome size is 9.93 Mbp and the *in-silico* G+C content 67.0%. The genome is rich in biosynthetic gene clusters predicted to encode for new specialised metabolites, notably antibiotics, and with genes with the capacity to produce products that promote plant growth.

The type strain ncl2^T^ (DSM 110931^T^ = CECT 30122^T^) was isolated from a root nodule of *Alnus glutinosa* growing in Leazes Park, Newcastle upon Tyne, UK. The GenBank accession number for the 16S rRNA gene and whole genome sequence of the strain were MZ014381 and JAGPOX000000000, respectively.

## Conclusions

Novel endophytic nocardiae are being isolated from rhizospheric soil [19, 113], plant roots and stems [26] and from nodules of actinorhizal plants [28–30] as in the case of *N. alni*. Nodular tissues are rich in carbohydrates hence they are excellent habitats for bacteria, including actinobacteria [114, 115]. Filamentous actinobaceria are associated with actinorhizal and legume root nodules, notably novel *Micromonospora* species [116]. The present study suggests that nocardiae, like micromonosporae, have the potential to promote plant growth though ecophysiological studies are needed to establish their interactions with plants, notably their role in root nodules of actinorhizal plants.

## Supplementary Information

**Additional file 1:** **Table S1.** Fatty acids profiles of strain ncl2^T^ and the type strain of *N*. *vaccinii*, its closest phylogenomic neighbour. **Table S2.** Putative natural product biosynthetic gene clusters detected in the draft genome of strain ncl2^T^ and *N*. *vaccinii *NBRC 15922^T^ using Antismash 5.0.0 webserver. **Figure S1.** Culture of isolate ncl2^T^ grown on medium DSMZ 65 after 10 days incubation at 28°C. **Figure S2.** Statistical comparisons of genomic properties of: 1, strain ncl2^T^, 2, *N*. *vaccinii *NBRC 15922^T^, 3, *N*. *casuarinae* BMG51109^T^, 4, *N*. *jiangxiensis* NBRC 101359^T^, 5, *N*. *miyunensis* NBRC 108239^T^ and 6, *N*. *pseudobrasiliensis* DSM 44290^T^. **Figure S3.** Frequency plot of POGs showing major COG categories. **Figure S4.** Functional categories found in the (a) COG/EggNog and (b) SEED analyses. **Figure S5.** Two-dimensional TLC plates of polar lipids extracted from isolate ncl2T (a) and *N*. *vaccinii* (b) stained with molybdatophosphoric acid (SigmaP1518). Key: DPG, diphosphatidylglycerol; PE, phosphatidylethanolamine; PG, phosphatidylglycerol;PI, phosphatidylinositol; GPL, glycophospholipid; AL1‐2, aminoplipids, GL1‐5, glycolipids and L1‐3, lipid. Solvent1: chloroform: methanol: distilled water (65:25:4); solvent 2: chloroform: glacial acetic acid: methanol: distilled water (80:12:15:4).

**Additional file 2:** **Table S3.** Pairwise ortholog matrix between the genome sequences of isolate ncl2^T^ (1) and *N*. *vaccinii* NBRC 15922^T^ (2), *N. casuarinae* BMG51109^T^ (3), *N*. *jiangxiensis* NBRC 101359^T^ (4), *N*. *miyunensis *NBRC 108239^T^ (5) and *N*. *pseudobrasiliensis* DSM 44290 ^T^ (6).

## Data Availability

The 16S rRNA gene and whole genome sequence of strain ncl2^T^ are available in the GenBank under accession numbers MZ014381 and JAGPOX000000000, respectively.
