# Supplementary material for: Genome insights into the pharmaceutical and plant growth promoting features of the novel species Nocardia alni sp. nov
Source: BMC Genomics. 2022 Jan 21;23:70. doi: 10.1186/s12864-021-08257-y (PMC8783487; doi:10.1186/s12864-021-08257-y)
Supplement: Supplementary file 1 — Additional file 1: Table S1. Fatty acids profiles of strain ncl2T and the type strain of N. vaccinii, its closest phylogenomic neighbour. Table S2. Putative natural product biosynthetic gene clusters detected in the draft genome of strain ncl2T and N. vaccinii NBRC 15922T using Antismash 5.0.0 webserver. Figure S1. Culture of isolate ncl2T grown on medium DSMZ 65 after 10 days incubation at 28°C. Figure S2. Statistical comparisons of genomic properties of: 1, strain ncl2T, 2, N. vaccinii NBRC 15922T, 3, N. casuarinae BMG51109T, 4, N. jiangxiensis NBRC 101359T, 5, N. miyunensis NBRC 108239T and 6, N. pseudobrasiliensis DSM 44290T. Figure S3. Frequency plot of POGs showing major COG categories. Figure S4. Functional categories found in the (a) COG/EggNog and (b) SEED analyses. Figure S5. Two-dimensional TLC plates of polar lipids extracted from isolate ncl2T (a) and N. vaccinii (b) stained with molybdatophosphoric acid (SigmaP1518). Key: DPG, diphosphatidylglycerol; PE, phosphatidylethanolamine; PG, phosphatidylglycerol;PI, phosphatidylinositol; GPL, glycophospholipid; AL1‐2, aminoplipids, GL1‐5, glycolipids and L1‐3, lipid. Solvent1: chloroform: methanol: distilled water (65:25:4); solvent 2: chloroform: glacial acetic acid: methanol: distilled water (80:12:15:4). [file 12864_2021_8257_MOESM1_ESM.docx]

**Genome insights into the pharmaceutical and the plant growth promoting features of the novel species *Nocardia alni* sp. nov.**

Imen Nouioui^1*^, Sung-min Ha^2^, Inwoo Baek^3^, Jongsik Chun^3,4^, Michael Goodfellow^5^

^1^Department of Microorganisms, Leibniz Institute DSMZ–German Collection of Microorganisms and Cell Cultures, 38124 Braunschweig, Germany

^2^Department of Integrative Biology and Physiology, University of California, Los Angeles, 610 Charles E. Young Drive East, Los Angeles, CA 90095, USA

^3^School of Biological Sciences & Institute of Molecular Biology and Genetics, Seoul National University, Seoul, Korea

^4^ChunLab, Inc., Seoul, Korea

^5^School of Natural and Environmental Sciences, Newcastle University, Ridley Building 2, Newcastle upon Tyne, NE1 7RU, United Kingdom

**Corresponding author**: Imen Nouioui, email: [imen.nouioui@dsmz.de](mailto:imen.nouioui@dsmz.de), Tel. ++49531-2616-369, Fax. ++49531-2616-418

**Supplementary table and figure legends**

**Table S1.** Fatty acids profiles of strain ncl2^T^ and the type strain of *N. vaccinii,* its closest phylogenomic neighbour.

**Table S2**. Putative natural product biosynthetic gene clusters detected in the draft genome of strain ncl2^T^ and *N. vaccinii* NBRC 15922^T^ using Antismash 5.0.0 webserver.

**Table S3.** Pairwise ortholog matrix between the genome sequences of isolate ncl2^T^ (1) and *N. vaccinii* NBRC 15922^T^ (2), *N. casuarinae* BMG51109^T^ (3)*, N. jiangxiensis* NBRC 101359^T^ (4)*, N. miyunensis* NBRC 108239^T^ (5) and *N. pseudobrasiliensis* DSM 44290 ^T^ (6)

**Figure S1.** Culture of isolate ncl2^T^ grown on medium DSMZ 65 after 10 days incubation at 28°C

**Figure S2.** Statistical comparisons of genomic properties of: 1, strain ncl2^T^, 2, *N. vaccinii* NBRC 15922^T^*,* 3, *N. casuarinae* BMG51109^T^*,* 4, *N. jiangxiensis* NBRC 101359^T^*,* 5, *N. miyunensis* NBRC 108239^T^ and 6, *N. pseudobrasiliensis* DSM 44290 ^T^

**Figure S3.** Frequency plot of POGs showing major COG categories

**Figure S4**. Functional categories found in the (a) COG/EggNog and (b) SEED analyses

**Figure S5.** Two-dimensional TLC plates of polar lipids extracted from isolate ncl2^T^ (a) and *N. vaccinii* (b) stained with molybdatophosphoric acid (SigmaP1518). Key: DPG, diphosphatidylglycerol; PE, phosphatidylethanolamine; PG, phosphatidylglycerol;PI, phosphatidylinositol; GPL, glycophospholipid; AL1‐2, aminoplipids, GL1‐5, glycolipids and L1‐3, lipid. Solvent1: chloroform: methanol: distilled water (65:25:4); solvent 2: chloroform: glacial acetic acid: methanol: distilled water (80:12:15:4).

**Table S1.** Fatty acids profiles of strain ncl2 and the type strain of *N. vaccinii,* its closest phylogenomic neighbour

| **Fatty acids** | **Strain ncl2^T^** | ***N. vaccinii* DSM 43285^T^** | ***Nocardia jiangxiensis* 43401^T*^** | ***Nocardia miyunensis* 117^T*^** |
| --- | --- | --- | --- | --- |
| C_16 :0_ | 39.2 | 37.3 | 24.8 | 27.2 |
| C_16 : 1_ ω7c | - | - | 12.4 | 16.4 |
| C_17 :1_ ω9c | 6.0 | - | - | - |
| C_17 :1_ ω5c | 4.3 | 4.0 | - | - |
| C_18 :0_ | - | - | 11.0 | 6.4 |
| C_18 :1_ω9c | 21.4 | 9.6 | 12.3 | 7.7 |
| C_18 :0_ 10-methyl | 9 .8 | 21.9 | 27.3 | 33.0 |
| Summed feature 3 C_16 :1_ω7c/C_16 :1_ω6c | 5 .4 | 8.1 | - | - |

Only fatty acids > 4 % are considered. -, fatty acid is absent; ^*^ fatty acid data taken from Cui et al. 2005

**Table S2**. Putative natural product biosynthetic gene clusters detected in the draft genome of strain ncl2^T^ and *N. vaccinii* NBRC 15922^T^ using Antismash 5.0.0 webserver

| **Isolate ncl2^T^** | | | | | |
| --- | --- | --- | --- | --- | --- |
| Type | From | To | Most similar known cluster | | Similarity |
| NRPS,RiPP-like | 102,337 | 129,534 | himastatin | NRP | 8% |
| T1PKS | 1 | 27,953 | tylactone | Polyketide | 15% |
| NRPS | 1 | 39,752 | A54145 | NRP | 5% |
| T1PKS | 80,213 | 109,041 | amycolamycin A / amycolamycin B | Polyketide | 2% |
| T1PKS | 50,577 | 90,968 | tiacumicin B | Polyketide:Modular type I | 6% |
| NRPS | 42,582 | 87,073 |  |  |  |
| T1PKS | 43,009 | 86,311 | teicoplanin | NRP:Glycopeptide | 3% |
| NRPS | 12,703 | 75,131 | fengycin | NRP | 13% |
| NRPS,redox-cofactor | 3,792 | 70,004 | JBIR-06 | NRP + Polyketide | 22% |
| NRPS,T1PKS | 1 | 42,636 | echoside A / echoside B / echoside C / echoside D / echoside E | NRP | 11% |
| terpene | 50,627 | 64,994 |  |  |  |
| NAPAA | 29,17 | 61,195 | steffimycin D | Polyketide:Type II + Saccharide:Hybrid/tailoring | 8% |
| T1PKS | 19,086 | 60,181 |  |  |  |
| NRPS | 1 | 59,712 |  |  |  |
| betalactone | 51 | 31,832 | lasalocid | Polyketide | 3% |
| T1PKS | 25,255 | 56,309 |  |  |  |
| terpene | 39,132 | 56,213 | 2-methylisoborneol | Terpene | 75% |
| NRPS | 1 | 48,334 | atratumycin | NRP | 7% |
| terpene | 13,256 | 34,311 | carotenoid | Terpene | 18% |
| T1PKS | 1 | 53,857 | nystatin | Polyketide | 31% |
| terpene | 29,28 | 48,28 |  |  |  |
| other | 1 | 29,939 | capreomycin IA / capreomycin IB / capreomycin IIA / capreomycin IIB | NRP | 6% |
| arylpolyene | 1,68 | 42,858 |  |  |  |
| indole | 1 | 17,652 |  |  |  |
| terpene | 1,656 | 23,659 |  |  |  |
| linaridin | 3,174 | 23,707 |  |  |  |
| NRPS,T1PKS | 1 | 35,298 |  |  |  |
| ectoine | 10,575 | 20,964 | ectoine | Other | 100% |
| NRPS | 1 | 23,103 | lasalocid | Polyketide | 3% |
| terpene | 16,554 | 27,793 |  |  |  |
| NRPS,terpene | 1 | 24,845 |  |  |  |
| NRPS | 1 | 24,782 |  |  |  |
| terpene | 1 | 19,541 | prejadomycin / rabelomycin / gaudimycin C / gaudimycin D / UWM6 / gaudimycin A | Polyketide:Type II + Saccharide:Hybrid/tailoring | 4% |
| NRPS | 1 | 21,043 | pepticinnamin E | NRP + Polyketide | 6% |
| T1PKS | 1 | 17,15 | stambomycin A / stambomycin B / stambomycin C / stambomycin D | Polyketide:Modular type I + Saccharide:Hybrid/tailoring | 36% |
| lassopeptide | 1 | 5,329 | anantin B1 / anantin B2 | RiPP | 40% |

| ***N. vaccinii* NBRC 15922^T^** | | | | |  |
| --- | --- | --- | --- | --- | --- |
| Type | From | To | Most similar known cluster | | Similarity |
| T1PKS | 91,036 | 137,032 |  |  |  |
| T1PKS | 9,243 | 45,775 |  |  |  |
| NRPS,RiPP-like | 334,57 | 434,306 | himastatin | NRP | 8% |
| terpene | 258,811 | 279,782 | isorenieratene | Terpene | 28% |
| lanthipeptide-class-i | 356,921 | 381,5 |  |  |  |
| ectoine | 403,687 | 414,082 | ectoine | Other | 100% |
| NAPAA | 39,654 | 73,577 | steffimycin D | Polyketide:Type II + Saccharide:Hybrid/tailoring | 8% |
| NRPS | 147,202 | 205,255 |  |  |  |
| betalactone | 304,683 | 332,993 |  |  |  |
| NRPS | 2,512 | 56,659 | tetarimycin A / tetarimycin B | Polyketide:Type II | 8% |
| terpene | 104,024 | 124,968 | carotenoid | Terpene | 18% |
| arylpolyene | 1 | 26,582 |  |  |  |
| terpene,betalactone | 48,68 | 81,44 | leinamycin | NRP + Polyketide:Modular type I + Polyketide:Trans-AT type I | 2% |
| lanthipeptide-class-i | 4,122 | 28,373 | nisin O | RiPP | 26% |
| terpene | 35,54 | 56,631 | A54145 | NRP | 5% |
| T3PKS | 44,495 | 85,607 | teicoplanin | NRP:Glycopeptide | 3% |
| lanthipeptide-class-v,NRPS,T1PKS,terpene | 134,618 | 305,416 | cremimycin | Polyketide | 5% |
| NRPS,T1PKS | 5,205 | 73,19 | cyphomycin | Polyketide | 2% |
| NRPS | 78,757 | 132,454 | retimycin A | NRP:Cyclic depsipeptide | 13% |
| betalactone | 12,164 | 34,959 |  |  |  |
| NRPS | 8,925 | 44,376 |  |  |  |
| T1PKS | 1 | 23,973 |  |  |  |
| terpene | 6,748 | 27,731 | isorenieratene | Terpene | 57% |
| T1PKS | 1 | 37,162 | caniferolide A / caniferolide B / caniferolide C / caniferolide D | Polyketide:Modular type I | 6% |
| redox-cofactor | 29,467 | 52,179 |  |  |  |
| NRPS | 54,633 | 90,449 |  |  |  |
| terpene | 67,989 | 81,644 |  |  |  |
| NRPS | 1 | 39,616 |  |  |  |
| NRPS | 1 | 48,508 | rustmicin | Polyketide:Iterative type I | 6% |


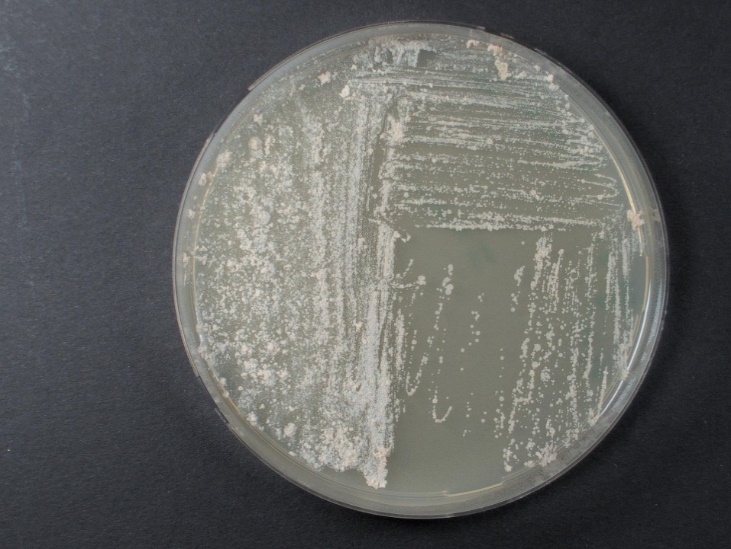


**Figure S1.** Culture of isolate ncl2^T^ grown on medium DSMZ 65 after 10 days of incubation at 28°C.


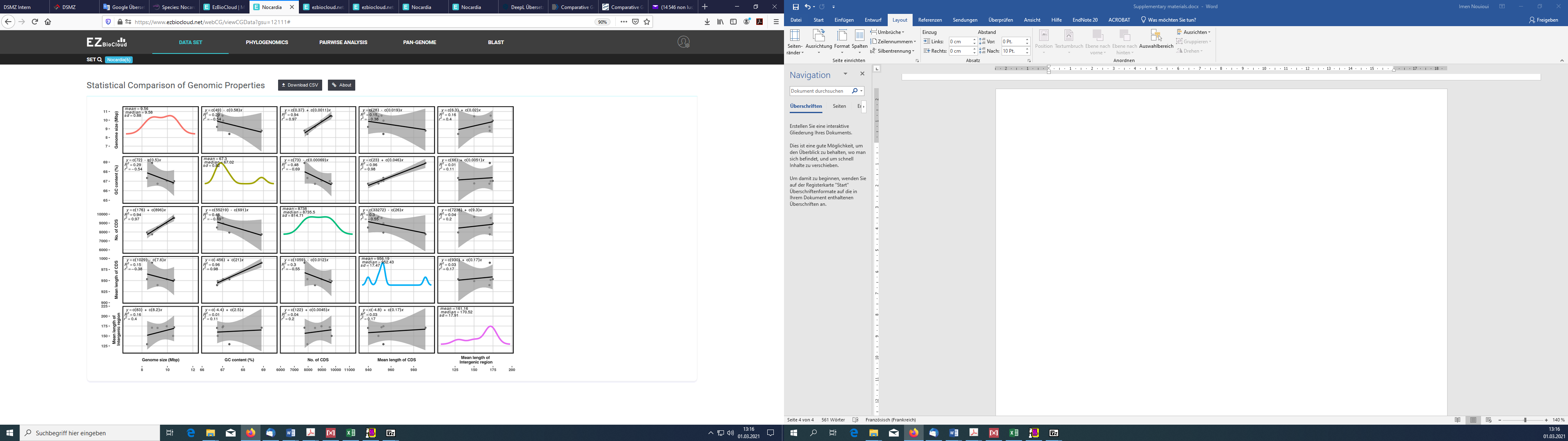


**Figure S2.** Statistical comparisons of genomic properties of: 1, strain ncl2^T^, 2, *N. vaccinii* NBRC 15922^T^*,* 3, *N. casuarinae* BMG51109^T^*,* 4, *N. jiangxiensis* NBRC 101359^T^*,* 5, *N. miyunensis* NBRC 108239^T^ and 6, *N. pseudobrasiliensis* DSM 44290 ^T^.


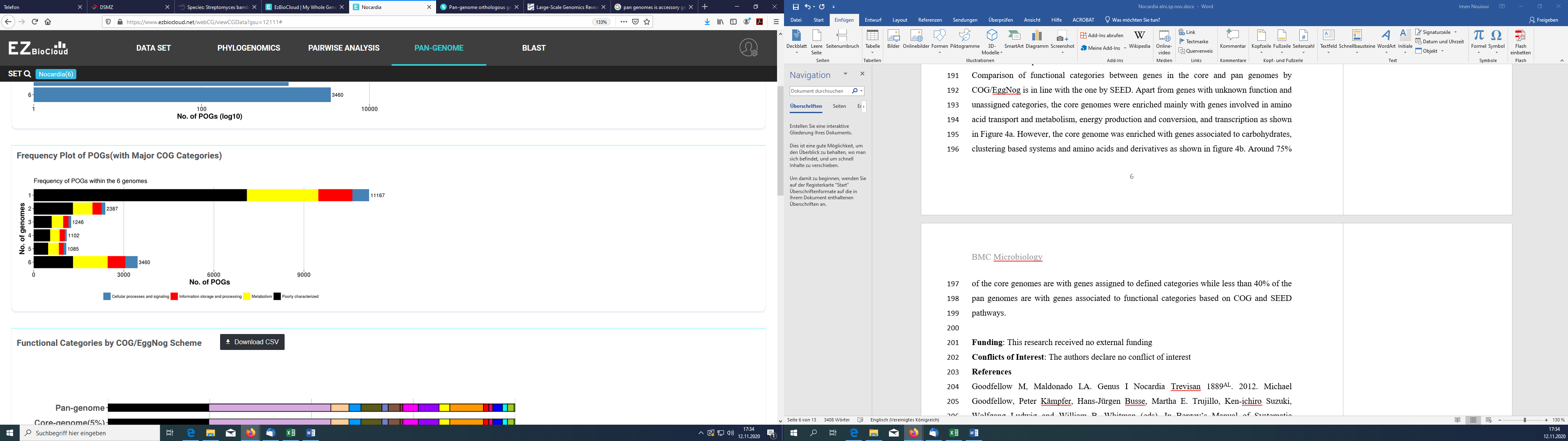


**Figure S3.** Frequency plot of POGs showing major COG categories. 1, strain ncl2^T^, 2, *N. vaccinii* NBRC 15922^T^*,* 3, *N. casuarinae* BMG51109^T^*,* 4, *N. jiangxiensis* NBRC 101359^T^*,* 5, *N. miyunensis* NBRC 108239^T^ and 6, *N. pseudobrasiliensis* DSM 44290 ^T^.


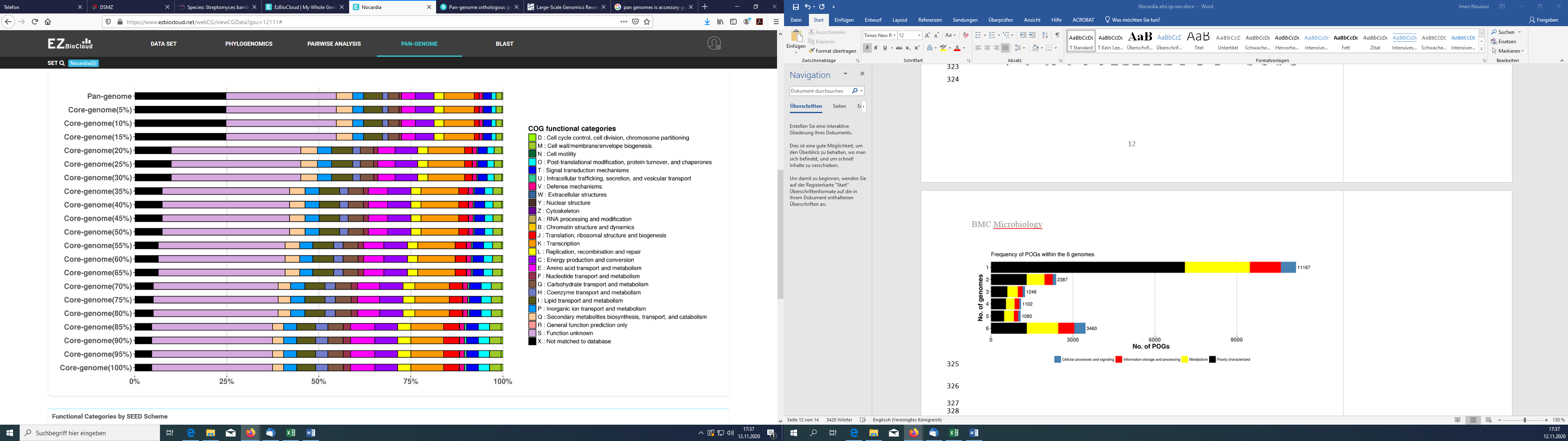


**a**


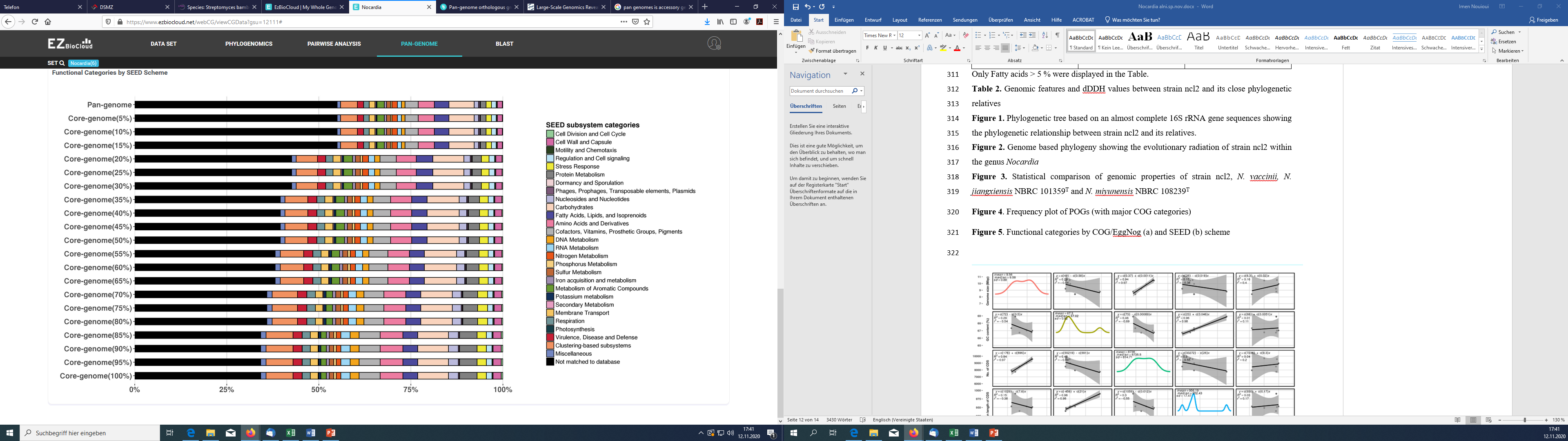


**b**

**Figure S4**. Functional categories found in the (a) COG/EggNog and (b) SEED analyses

**Figure S5.** Two-dimensional TLC plates of polar lipids extracted from isolate ncl2^T^ (a) and *N. vaccinii* DSM 43285^T^(b) stained with molybdatophosphoric acid (SigmaP1518). Key: DPG, diphosphatidylglycerol; PE, phosphatidylethanolamine; PI, phosphatidylinositol; PGL, phosphoglycolipid; AL, aminoplipid, GL, glycolipid, L1‐2, lipids and PL1-2, phospholipids. Solvent1: chloroform: methanol: distilled water (65:25:4 v/v/v/); solvent 2: chloroform: glacial acetic acid: methanol: distilled water (80:12:15:4 v/v/v).
